# Biallelic *RSPH4A* loss-of-function variants cause primary ciliary dyskinesia in a Chinese patient

**DOI:** 10.3389/fgene.2026.1857456

**Published:** 2026-07-08

**Authors:** Yu-Ting Lu, Hui-Yan Tang, Kai Chen, Ding-Yuan Lai, De-Cheng Wang, Fan Yang

**Affiliations:** 1 Department of Pediatrics, Jiaxing Hospital of Traditional Chinese Medicine Affiliated to Zhejiang Chinese Medical University, Jiaxing, China; 2 The Research Center for Lin He Academician New Medicine, Institutes for Shanghai Pudong Decoding Life, Shanghai, China; 3 Lishui Key Laboratory of Brain Health and Severe Brain Disorders, Lishui Second People’s Hospital, Wenzhou Medical University, Lishui, China; 4 Bio-X Institutes, Key Laboratory for the Genetics of Developmental and Neuropsychiatric Disorders, Ministry of Education, Shanghai Jiao Tong University, Shanghai, China

**Keywords:** functional validation, genetic counseling, loss-of-function variants, primary ciliary dyskinesia, RSPH4A, whole exome sequencing

## Abstract

**Background:**

Primary ciliary dyskinesia (PCD) is a rare autosomal recessive disorder characterized by defective motile cilia function, affecting approximately one in 7,500 to one in 10,000 live births. Pathogenic variants in radial spoke head genes, including *RSPH4A*, cause PCD with distinctive central-microtubular-pair defects. However, the functional consequences of novel *RSPH4A* variants remain poorly characterized, limiting genetic counseling and prenatal diagnostic capabilities. This study aims to reveal the genetic etiology of PCD in an affected family and the clinical significance of the two novel *RSPH4A* variants identified in this PCD-affected pedigree.

**Methods:**

We recruited a five-member Chinese family including an 11-year-old female PCD proband presenting with chronic bronchiectasis and recurrent respiratory infections. Comprehensive clinical evaluations, whole exome sequencing (WES), and Sanger sequencing were performed to identify genetic variants. Bioinformatics analyses including protein sequence alignment and structural modeling were conducted. Experimental validation employed site-directed mutagenesis, quantitative real-time PCR, and Western blotting in HEK293T cells to characterize variant effects on mRNA stability and protein expression.

**Results:**

WES identified compound heterozygous *RSPH4A* variants in the proband: *RSPH4A* (NM_001010892.3): c.2T>C (p.Met1Thr) inherited from the mother and *RSPH4A* (NM_001010892.3): c.854delA (p.Lys286Serfs*22) inherited from the father, showing strict co-segregation with the disease phenotype. The c.2T>C variant disrupted the translation initiation codon, while c.854delA introduced a premature termination codon within the radial spoke head domain. Cross-species analysis demonstrated high conservation of the affected region across 15 vertebrate species. Structural modeling predicted complete loss of the radial spoke head domain in the truncated protein. Functional studies revealed that c.2T>C severely impaired protein translation despite intact mRNA levels, whereas c.854delA triggered nonsense-mediated mRNA decay and produced unstable truncated protein. Both variants resulted in substantial reduction in functional RSPH4A protein expression.

**Conclusion:**

This study identifies novel loss-of-function *RSPH4A* variants causing PCD through distinct molecular mechanisms, expanding the mutational spectrum of radial spoke head protein-related ciliopathies. These findings offer compelling proof in favor of a molecular diagnosis of PCD in this family and enable carrier screening for at-risk relatives. The experimental validation strategy establishes a framework for interpreting variants of uncertain significance in PCD genes, facilitating accurate prenatal diagnosis and preimplantation genetic testing to reduce the recurrence risk and birth defect incidence in PCD. Furthermore, understanding the precise functional consequences of *RSPH4A* variants informs genotype-phenotype correlations and may guide future development of targeted therapeutic interventions for this debilitating respiratory disorder.

## Highlights

- Novel biallelic *RSPH4A* loss-of-function variants identified in Chinese PCD patient;
- *RSPH4A* (NM_001010892.3): c.2T>C (p.Met1Thr) disrupts translation initiation; *RSPH4A* (NM_001010892.3): c.854delA (p.Lys286Serfs*22) triggers nonsense-mediated mRNA decay (NMD);
- Functional validation enables genetic counseling and prenatal diagnosis for PCD.

## 1 Introduction

Primary ciliary dyskinesia (PCD; MIM #244400) is a rare, genetically heterogeneous autosomal recessive disorder characterized by defective structure and/or function of motile cilia (Wee et al., 2024; Despotes et al., 2024; Guan et al., 2021). Recent population-based studies estimate the prevalence of PCD at approximately one in 7,500 to one in 10,000 live births worldwide, though these figures likely represent underestimates due to under-recognition and diagnostic challenges (Despotes et al., 2024; Hannah et al., 2022). The clinical spectrum encompasses neonatal respiratory distress, chronic oto-sino-pulmonary infections with progressive bronchiectasis, and laterality defects occurring in approximately 50% of patients (Zurcher et al., 2021; Kapur et al., 2009; Shoemark et al., 2025). These laterality defects include situs inversus totalis (complete mirror-image reversal of thoracic and abdominal organs), dextrocardia, and heterotaxy syndromes (Shoemark et al., 2025). The subset of PCD patients with situs inversus totalis, chronic sinusitis, and bronchiectasis is collectively termed Kartagener’s syndrome (Zurcher et al., 2021; Kapur et al., 2009). Affected individuals also frequently present with chronic rhinosinusitis, persistent otitis media with hearing impairment, and male infertility resulting from impaired sperm motility (Mener et al., 2013; L et al., 2015; Mata et al., 2014; Sironen et al., 2020). The pathophysiological basis of PCD stems from dysfunctional mucociliary clearance due to abnormal ciliary beat patterns or ultrastructural defects, particularly involving outer dynein arms (ODA), inner dynein arms (IDA), and central apparatus abnormalities (Despotes et al., 2024; Pereira et al., 2023). Diagnosis remains challenging due to the absence of a single gold standard test, requiring a combination of clinical evaluation, nasal nitric oxide (nNO) measurement, high-speed video microscopy analysis (HSVMA), transmission electron microscopy (TEM), and genetic testing (Mirra et al., 2017; Shapiro et al., 2016; Keicho et al., 2023; Snijders et al., 2014). Notably, the median age at diagnosis in European cohorts is estimated at 5.0–6.8 years (Kuehni et al., 2010), with many patients experiencing considerable diagnostic delays and progressive lung damage prior to definitive diagnosis (Shoemark et al., 2025; Lucas et al., 2017). This diagnostic delay is attributed to the nonspecific nature of early symptoms and limited awareness of PCD among primary care physicians (Shapiro et al., 2016; Kuehni et al., 2010). In the Chinese population, PCD remains significantly underdiagnosed, and comprehensive epidemiological data are still emerging, highlighting the urgent need for improved genetic diagnosis in this region (Peng et al., 2022; Guo et al., 2020; Li et al., 2022; Zhang et al., 2025).

The genetic architecture of PCD involves biallelic pathogenic variants in more than 50 genes encoding structural components, assembly factors, and regulatory proteins essential for ciliary function (Horani and Ferkol, 2018; Knowles et al., 2013; Zhao et al., 2021). Among these, radial spoke head proteins play critical roles in regulating ciliary motility by transmitting signals from the central pair complex to dynein arms, thereby governing waveform and bend direction (Grossman-Haham et al., 2021; Gui et al., 2021). The radial spoke head protein 4 homolog A (*RSPH4A*; MIM #612647) encodes a key component of the radial spoke head complex, which is essential for maintaining the structural integrity of the central pair microtubules and coordinating ciliary beating (Yoke et al., 2020). Pathogenic variants in *RSPH4A* were first identified by Castleman et al. (2009), who demonstrated that loss-of-function mutations cause PCD with distinctive central-microtubular-pair abnormalities observable by TEM (Castleman et al., 2009). Unlike dynein arm defects, *RSPH4A*-related PCD is characterized by the absence of laterality defects, as nodal cilia possess a 9 + 0 configuration lacking the central pair apparatus that is affected by radial spoke dysfunction (Castleman et al., 2009). Cryo-electron tomography studies have further elucidated that RSPH4A is critical for assembly of the triplet radial spoke (RS) heads RS1, RS2, and RS3, explaining the severe motility defects observed in patients despite partially preserved ciliary ultrastructure (Grossman-Haham et al., 2021; Lin et al., 2014; Meng et al., 2024). To date, more than 30 pathogenic variants have been associated with *RSPH4A*-related PCD, including the well-characterized founder mutation c.921 + 3_921+6delAAGT in Puerto Rican Hispanics and diverse mutations in other populations (Castleman et al., 2009; De Jesus-Rojas et al., 2022; Wang et al., 2022; Feng et al., 2023; De Jesus-Rojas et al., 2023).

Whole exome sequencing (WES) has emerged as a powerful diagnostic tool for PCD, with reported genetic diagnostic yields ranging from 30% to 76% depending on the population, clinical selection criteria, and analytical approach (Guan et al., 2021; Hannah et al., 2022; Oh et al., 2024; Gileles-Hillel et al., 2020). Large-scale studies have demonstrated that WES provides diagnostic rates of approximately 30%–50% in patients with suspected rare Mendelian disorders, significantly higher than conventional targeted testing approaches (Han et al., 2025; Dillon et al., 2018; Trujillano et al., 2017). The identification of novel pathogenic variants continues to expand the mutational spectrum and improve our understanding of genotype-phenotype correlations (Castleman et al., 2009). In this study, we report a Chinese PCD patient presenting with classic respiratory manifestations in whom WES identified two novel variants in *RSPH4A* (NM_001010892.3): c.2T>C (p.Met1Thr), disrupting the initiation codon, and c.854delA (p.Lys286Serfs*22), causing a frameshift and premature termination. Both variants are absent from population databases and predicted to result in substantial decrease in functional RSPH4A protein through distinct molecular mechanisms. We performed comprehensive functional characterization including quantitative real-time PCR (qRT-PCR), Western blotting (WB) analysis, protein structural modeling, and evolutionary conservation analysis to demonstrate that these variants are indeed loss-of-function mutations. This study expands the known mutational spectrum of *RSPH4A*, provides insights into the functional consequences of start codon and frameshift variants, and underscores the importance of WES combined with functional validation in establishing the genetic diagnosis of PCD.

## 2 Methods and materials

### 2.1 Patient recruitment and ethical approval

This study enrolled a five-member Chinese family consisting of a female proband (11 years old) diagnosed with PCD, her unaffected parents, her elder sister (19 years old), and her dizygotic twin sister (11 years old). The proband was admitted to the Department of Pediatrics, Jiaxing Hospital of Traditional Chinese Medicine (T.C.M.) (Zhejiang Province, China), presenting with chief complaints of “recurrent cough and sputum production for 4 years, aggravated for 1 week”. The family was recruited through the pediatric respiratory clinic following standardized recruitment protocols for rare genetic diseases.

Prior to enrollment, written informed consent was obtained from all participants or their legal guardians (parents for the proband and her twin sister). The study protocol was reviewed and approved by the Ethics Committee of Jiaxing Hospital of T.C.M. (Approval No. JXHTCM-EC-2024-029) and conducted in accordance with the Declaration of Helsinki principles. All clinical investigations were performed following standardized procedures, and participants were informed of their right to withdraw from the study at any time without affecting their medical care.

### 2.2 Clinical evaluation and diagnostic workup

Comprehensive clinical assessments were performed on the proband to establish the PCD diagnosis and characterize disease severity: (1) Physical examination included measurements of height, weight, body temperature, pulse rate, respiratory rate, blood pressure, peripheral oxygen saturation (SpO2), mental status, and systematic examination of skin, nasal passages, and tonsillar region; (2) Laboratory investigations comprised complete blood count (white blood cells, neutrophils, lymphocytes, eosinophils, hemoglobin, and platelets), high-sensitivity C-reactive protein (hs-CRP), liver and renal function tests, cardiac enzyme panels, coagulation profiles, procalcitonin, ferritin, lymphocyte subpopulation analysis, tuberculosis antibody testing, purified protein derivative (PPD) skin test, *Mycoplasma* pneumoniae nucleic acid detection, and serum immunoglobulin levels (IgG, IgA, and IgM); (3) Cardiac evaluation included standard 12-lead electrocardiography (ECG) and transthoracic echocardiography to exclude congenital heart defects; (4) Allergen screening was performed using comprehensive allergen panels including house dust mites (*Dermatophagoides pteronyssinus* and *Dermatophagoides farinae*) and rhinovirus RNA detection from nasal swabs; (5) Imaging studies consisted of high-resolution computed tomography (HRCT) of the paranasal sinuses and chest to assess for bronchiectasis, sinusitis, and situs abnormalities; (6) Audiological assessment included pure-tone audiometry and tympanometry to evaluate chronic otitis media; (7) Nasal nitric oxide (nNO) measurement was conducted using chemiluminescence analyzers (NIOX MINO, Aerocrine AB, Sweden) following international guidelines, with nNO levels <77 nL/min considered abnormal; and (8) Pulmonary function testing included bronchodilator reversibility testing using spirometry to assess airway obstruction.

### 2.3 Biospecimen collection and processing

Peripheral venous blood samples (approximately 2 mL per individual) were collected from all five family members using BD Vacutainer EDTA tube (BD Biosciences, Cat. No. 367856). Blood collection was performed by trained phlebotomists following standard aseptic techniques. Prior to collection, detailed informed consent was obtained regarding the use of biological materials for genetic research.

Blood samples were processed within 4 h (hrs) of collection. Peripheral blood mononuclear cells (PBMCs) were isolated by density gradient centrifugation using Ficoll-Paque PLUS (Cytiva, Cat. No. 17144002) at 400×g for 30 min (min) at room temperature (RT). The buffy coat layer containing leukocytes was carefully aspirated and washed twice with phosphate-buffered saline (PBS, ThermoFisher Scientific, Cat. No. 10010023). Genomic DNA extraction was performed using the QIAamp DNA Blood Mini Kit (Qiagen, Cat. No. 51104) according to the manufacturer’s protocol. DNA concentration and purity were assessed using a NanoDrop One spectrophotometer (ThermoFisher Scientific, United States), with samples showing A260/A280 ratios between 1.8-2.0 and A260/A230 ratios >1.5 considered acceptable for downstream applications. DNA integrity was verified by 1.0% agarose gel electrophoresis. Extracted DNA was stored at −20 °C until further analysis.

### 2.4 Whole exome sequencing and Sanger Sequencing Validation

Library Preparation and Sequencing: WES was performed on the proband’s genomic DNA. Briefly, 1.0 μg of high-quality genomic DNA was sheared using Covaris S220 Focused-ultrasonicator (Covaris, Inc.) to generate 150–200 bp fragments. DNA libraries were constructed using the SureSelectXT Human All Exon V6 kit (Agilent Technologies, Cat. No. 5190–8863) following the manufacturer’s instructions. This kit targets approximately 60 Mb of coding regions (including mitochondrial genes) and known disease-associated non-coding regions. Adapter-ligated libraries were amplified by PCR using Herculase II Fusion DNA Polymerases (Agilent Technologies, Cat. No. 600675) and purified using AMPure XP beads (Beckman Coulter, Cat. No. A63881). Library quality was assessed using the 2100 Bioanalyzer (Agilent Technologies) with High Sensitivity DNA Analysis Kit (Cat. No. 5067-4626), ensuring library insert sizes of 200–300 bp and concentrations >2 nM.

High-Throughput Sequencing: Cluster generation was performed on the cBot system (Illumina, Inc.) followed by paired-end sequencing (2 × 150 bp) on the NovaSeq 6000 platform (Illumina, Inc.) using NovaSeq 6000 S4 Reagent Kit v1.5. A minimum of 15 Gb raw data per sample was generated to achieve >150 × mean coverage of the target regions, with >99% of target bases covered at ≥20× depth.

Bioinformatics Analysis: Raw sequencing data in FASTQ format underwent quality control using FastQC v0.11.9 (Babraham Bioinformatics) and MultiQC v1.9. Adapter sequences and low-quality reads (Phred score <20) were trimmed using Trimmomatic v0.39. Clean reads were aligned to the human reference genome GRCh37/hg19 using Burrows-Wheeler Aligner (BWA-MEM) v0.7.17 with default parameters. Duplicate reads were marked using Picard v2.23.3 (Broad Institute) and excluded from downstream analysis.

Local realignment around indels and base quality score recalibration was performed using Genome Analysis Toolkit (GATK) v4.1.9.0. Single nucleotide variants (SNVs) and insertions/deletions (indels) were called using GATK HaplotypeCaller with GVCF workflow, followed by joint genotyping using GenotypeGVCFs. Variant quality score recalibration (VQSR) was applied to filter variants based on known training sets from dbSNP, 1000 Genomes, and HapMap projects.

Variant annotation was performed using ANNOVAR (version 2018Apr16) with multiple databases including RefSeq, Ensembl, dbSNP v151, gnomAD v2.1.1, ExAC, 1000 Genomes Project, ClinVar, and OMIM. Functional impact prediction was conducted using SIFT (v6.2.1), PolyPhen-2 (v2.2.2), MutationTaster 2021, CADD (v1.6), and REVEL.

Variant Filtering Strategy: Variants were filtered using the following criteria: (1) minor allele frequency (MAF) <0.01 in gnomAD and ExAC databases; (2) located within PCD-associated genes (n = 50 genes including *RSPH4A*, *DNAH5*, *DNAH11*, *DNAI1*, *DNAI2*, and *TXNDC3*, etc.); (3) predicted to be protein-altering (missense, nonsense, frameshift, splice-site, or in-frame indels); (4) consistent with autosomal recessive inheritance pattern. Compound heterozygous or homozygous variants were prioritized for validation.

Sanger Sequencing Validation: Candidate variants in *RSPH4A* (NM_001010892.3: c.2T>C and c.854delA) were validated in all five family members using Sanger sequencing. PCR primers were designed using Primer3Plus to flank the variant sites: for c.2T>C (Forward: 5′-CTA​TGG​AGA​TAG​GAC​GCA​GCA-3′, Reverse: 5′-CAG​GAG​CAG​GTG​ATG​ATG​TTT-3′); for c.854delA (Forward: 5′-TCT​GGA​ATT​TTC​TCT​GTT​TGG​ATA-3′, Reverse: 5′-AGC​CTG​GGA​GCC​TGT​CTA​AA-3′). PCR amplification was performed using Phusion High-Fidelity DNA Polymerase (ThermoFisher Scientific, Cat. No. F530 L) with the following conditions: initial denaturation at 98 °C for 30 s (sec), followed by 35 cycles of 98 °C for 10 s, 60 °C for 30 s, and 72 °C for 30 s, with final extension at 72 °C for 5 min. PCR products were purified using ExoSAP-IT^TM^ Express (ThermoFisher Scientific, Cat. No. 75001) and sequenced using BigDye^TM^ Terminator v3.1 Cycle Sequencing Kit (ThermoFisher Scientific, Cat. No. 4337455) on an ABI 3730xl DNA Analyzer (Applied Biosystems, Cat. No. A41046). Sequencing traces were analyzed using SeqMan Pro v17.2 (DNASTAR, Inc.) to confirm variant presence and segregation within the family.

### 2.5 RSPH4A protein sequence conservation analysis

Cross-species conservation analysis of the RSPH4A protein sequence was performed to assess the evolutionary importance of the mutated residues. Full-length RSPH4A protein sequences from 15 vertebrate species were retrieved from the NCBI Protein database: *Homo sapiens* (NP_001010892.1), *Gorilla gorilla gorilla* (XP_063563882.1), *Macaca fascicularis* (XP_005551713.3), *Mus musculus* (AAI38002.1), *Rattus norvegicus* (NP_001101099.2), *Dama* (KAM9676167.1), *Megaptera novaeangliae* (KAM9060485.1), *Lycaon pictus* (KAM8964044.1), *Molossus nigricans* (KAM7136833.1), *Panthera onca* (XP_060472447.1), *Tamandua tetradactyla* (XP_077018844.1), *Camelus bactrianus* (XP_010963768.3), *Castor canadensis* (XP_073931944.1), *Manis javanica* (XP_017530088.1), and *Vicugna pacos* (XP_006205746.1).

Multiple sequence alignment was performed using PRALINE multiple sequence alignment tool (https://zeus.few.vu.nl/programs/pralinewww/) with default parameters including BLOSUM62 substitution matrix, gap open penalty of 12, and gap extension penalty of 1. Secondary structure prediction was incorporated using PSIPRED. The alignment was visualized using Jalview v2.11.2.0, and conservation scores were calculated using the ConSurf server to identify highly conserved residues across species. The Met1 and Lys286 residues were specifically examined for their degree of conservation and structural context within the radial spoke head domain.

### 2.6 RSPH4A protein structure modeling

Three-dimensional structural modeling of wild-type and mutant RSPH4A proteins was performed to predict the functional impact of the c.854delA (p.Lys286Serfs*22) variant. Strucutral modeling of RSPH4A protein was conducted using SWISS-MODEL (https://swissmodel.expasy.org/) (Waterhouse et al., 2018; Bienert et al., 2017). For wild-type RSPH4A, residues 1-716 were modeled using the automated modeling pipeline with ProMod3 version 3.2.1. For the mutant protein (p.Lys286Serfs*22), the premature termination codon introduces a truncated protein of 306 amino acids with a novel C-terminal sequence of 21 residues before termination. The mutant model was constructed by terminating the chain at position 306 and modeling the novel frameshift-derived peptide using loop modeling algorithms in SWISS-MODEL.

### 2.7 In silico pathogenicity prediction

The pathogenicity of identified variants was assessed using multiple computational prediction tools to establish evidence for loss-of-function mechanisms. Variant c.2T>C (p.Met1Thr) and c.854delA (p.Lys286Serfs*22) was analyzed using: (1) SIFT (version 6.2.1); (2) MutationTaster 2021 (http://www.mutationtaster.org/); (3) LOFTEE (Loss-of-Function Transcript Effect Estimator, Ensembl VEP v104); (4) Start_Lost prediction; (5) PROVEAN (version 1.1.3); and (6) Frameshift prediction.

### 2.8 RSPH4A Gene Synthesis, site-directed mutagenesis, and vector construction

Gene Synthesis: The wild-type *RSPH4A* coding sequence (CDS, 2151 bp) was chemically synthesized and codon-optimized for mammalian expression by GENERAL BIOL Biotech Corporation (Chuzhou, China). The synthesized sequence was subcloned into the pUC57 vector using TA cloning methodology, creating the plasmid pUC57-*RSPH4A*-WT. The integrity of the insert was verified by restriction enzyme digestion and full-length Sanger sequencing.

Site-Directed Mutagenesis: Two mutant variants were generated from the wild-type template using the Q5 Site-Directed Mutagenesis Kit (New England Biolabs, Cat. No. E0554S) according to the manufacturer’s protocol.

For c.2T>C (p.Met1Thr), the following mutagenic primers were designed: Forward 5′-ACT​CAG​ATC​TCG​AGG​CCA​CCA​CGG​AGG​ACT​CAA​CCT​CCC​CGA​AGC​A-3′, Reverse 5′- TGC​TTC​GGG​GAG​GTT​GAG​TCC​TCC​GTG​GTG​GCC​TCG​AGA​TCT​GAG​T-3’. This introduces a T>C change at the first coding nucleotide, converting the initiation codon ATG (Met) to ACG (Thr).

For c.854delA (p.Lys286Serfs*22), primers were: Forward 5′- AAT​GAG​AAT​GAG​TTG​CTT​CCA​ACA​TAT​GAA​ATA​GCA​GAA​AGC​AAA​AGG​CTC​TTT​TTC​TCC​AGG​GAC​ATT​TGG​A-3′, Reverse 5′- ACA​ATA​TAA​TTC​ATT​TCC​AGA​CCC​AAG​ATC​TTT​CCC​CAG​AAG​CGG​CAT​CTT​TGG​ATT​GGG-3’. This deletes the adenine at cDNA position 854.

PCR conditions for mutagenesis included initial denaturation at 98 °C for 30 s, followed by 25 cycles of 98 °C for 10 s, 55 °C–65 °C (primer-specific) for 30 s, and 72 °C for 3.5 min, with final extension at 72 °C for 2 min. Parental methylated DNA was digested with DpnI (New England Biolabs, Cat. No. R0176S) at 37 °C for 1 h. The resulting plasmids (pUC57-*RSPH4A*-c.2T>C and pUC57-*RSPH4A*-c.854delA) were transformed into NEB 5-α Competent *E. coli* (New England Biolabs, Cat. No. C2987H) and plated on LB agar containing 100 μg/mL ampicillin (Sigma-Aldrich, Cat. No. A5354).

Colony PCR was performed using M13F/M13R primers to identify positive clones. Plasmid DNA from 3-5 positive colonies per variant was purified using the QIAprep Spin Miniprep Kit (Qiagen, Cat. No. 27106) and subjected to Sanger sequencing to confirm the presence of the intended mutation and absence of PCR-induced errors. Verified plasmids were then used for downstream cloning.

Expression Vector Construction: The wild-type and mutant *RSPH4A* CDS sequences were subcloned into the eukaryotic expression vector pIRES2-EGFP using restriction enzyme cloning. The inserts and vector were double-digested with XhoI (New England Biolabs, Cat. No. R0146S) and BamHI (New England Biolabs, Cat. No. R0136S) at 37 °C for 2 hrs. Digested products were purified using the QIAquick Gel Extraction Kit (Qiagen, Cat. No. 28706) and ligated using T4 DNA Ligase (New England Biolabs, Cat. No. M0202S) at 16 °C overnight.

Epitope Tagging Strategy:

1. For wild-type and c.2T>C mutant: A 3×FLAG tag (DYKDDDDK-G-DYKDDDDK-I-DYKDDDDK) was fused to the C-terminus of the CDS (excluding the stop codon) using overlapping PCR with primers containing the FLAG sequence. This allows detection of full-length protein if translation initiates from an alternative start codon in the c.2T>C mutant;
2. For c.854delA mutant: The 3×FLAG tag was fused to the N-terminus because the frameshift introduces a premature stop codon at position 307, truncating the protein before the C-terminus.

The final constructs (pIRES2-EGFP-*RSPH4A*-WT-3×FLAG, pIRES2-EGFP-*RSPH4A*-c.2T>C-3×FLAG, and pIRES2-EGFP-3×FLAG-*RSPH4A*-c.854delA) were verified by restriction mapping and full-insert Sanger sequencing using vector-specific and internal primers.

Endotoxin-Free Plasmid Preparation: Large-scale plasmid DNA was prepared using the EndoFree Plasmid Maxi Kit (Qiagen, Cat. No. 12362) according to the manufacturer’s protocol. Endotoxin levels were confirmed to be <0.1 EU/μg using the PYROGENT Plus Gel Clot LAL Assay (Lonza, Cat. No. N283-06). Plasmid DNA was resuspended in endotoxin-free TE buffer (10 mM Tris-HCl, 1 mM EDTA, pH 8.0) and stored at −20 °C until transfection.

### 2.9 Cell Culture, transfection, and functional assays

Cell Culture: Human embryonic kidney 293T (HEK293T) cells were selected as an initial heterologous expression system due to their high transfection efficiency and widespread use in assessing variant effects on gene expression. HEK293T cells (ATCC CRL-3216^TM^) were maintained in Dulbecco’s Modified Eagle Medium (DMEM, ThermoFisher Scientific, Cat. No. 11965092) supplemented with 10% fetal bovine serum (FBS, ThermoFisher Scientific, Cat. No. 10099141C) and 1% penicillin-streptomycin (ThermoFisher Scientific, Cat. No. 15140122) at 37 °C in a humidified atmosphere containing 5% CO_2_. Cells were passaged every 2–3 days using 0.25% Trypsin-EDTA (ThermoFisher Scientific, Cat. No. 25200056) and seeded at appropriate densities for experiments. *Mycoplasma* contamination was routinely tested using the MycoAlert *Mycoplasma* Detection Kit (Lonza, Cat. No. LT07-318) and confirmed negative prior to experiments.

Transient Transfection: HEK293T cells were seeded in sterile 6-well plates (Corning, Cat. No. 3516) at a density of 5 × 10^5^ cells per well (80% confluence at time of transfection). Four experimental groups were established: (1) Negative control (NC, cells were transfected with empty pIRES2-EGFP vector), (2) Wild-type (WT) *RSPH4A*, (3) c.2T>C mutant, and (4) c.854delA mutant. Each group consisted of three biological replicates.

Transfection was performed using Lipofectamine 3000 Transfection Reagent (ThermoFisher Scientific, Cat. No. L3000015) following the manufacturer’s recommended protocol. Briefly, 2.5 μg of plasmid DNA and 3.75 μL of Lipofectamine 3000 reagent were diluted separately in Opti-MEM^TM^ I Reduced Serum Medium (ThermoFisher Scientific, Cat. No. 31985062), mixed with P3000 Enhancer Reagent, incubated for 10–15 min at RT, and added dropwise to the cells. The medium was replaced with fresh complete DMEM after 6–8 hrs. Cells were harvested 48 hrs post-transfection for downstream analyses.

Quantitative Real-Time PCR (qRT-PCR): Total RNA was extracted using TRIzol^TM^ Reagent (ThermoFisher Scientific, Cat. No. 15596026CN) following the manufacturer’s protocol. RNA concentration and purity were assessed using NanoDrop One (A260/A280 ratios 1.9–2.1). First-strand cDNA synthesis was performed using the PrimeScript^TM^ RT Reagent Kit with gDNA Eraser (TaKaRa, Cat. No. RR047A) with 1 μg of total RNA in a 20 μL reaction volume.

qRT-PCR was conducted using TB Green Premix Ex Taq^TM^ II (TaKaRa, Cat. No. RR820A) on a QuantStudio^TM^ 5 Real-Time PCR System (Applied Biosystems, Cat. No. A28575). The following primers were used: *RSPH4A* Forward: 5′-GTC​CTT​GGA​GCC​CGC​AGT​CTA​G-3′, *RSPH4A* Reverse: 5′-GTC​CTG​TCC​GAC​TGA​GGT​GGT​G-3’ (amplicon 163 bp); *GAPDH* Forward: 5′-CGG​AGT​CAA​CGG​ATT​TGG​TCG​TAT-3′, *GAPDH* Reverse: 5′-AGC​CTT​CTC​CAT​GGT​GGT​GAA​GAC-3’ (amplicon 307 bp).

PCR conditions included initial denaturation at 95 °C for 30 s, followed by 40 cycles of 95 °C for 5 s and 60 °C for 30 sec. Melting curve analysis was performed to confirm specific amplification. Relative gene expression was calculated using the 2^(-ΔΔCt) method with *GAPDH* as the internal reference gene. Each sample was analyzed in technical triplicates.

Western blotting: HEK293T cells were lysed in RIPA buffer (ThermoFisher Scientific, Cat. No. 89900) supplemented with Halt^TM^ Protease Inhibitor Cocktail (ThermoFisher Scientific, Cat. No. 78430) and PhosSTOP^TM^ Phosphatase Inhibitor (Roche, Cat. No. 4906845001) for 30 min on ice. Lysates were centrifuged at 12,000×g for 15 min at 4 °C, and supernatants were collected. Protein concentration was determined using the Pierce^TM^ BCA Protein Assay Kit (ThermoFisher Scientific, Cat. No. 23225).

Equal amounts of protein (30 μg per lane) were separated by 10% SDS-PAGE using Mini-PROTEAN TGX^TM^ Precast Protein Gels (Bio-Rad, Cat. No. 4561034) and transferred to Immobilon-P PVDF membranes (Millipore, Cat. No. IPVH00010) using the Trans-Blot Turbo^TM^ Transfer System (Bio-Rad, Cat. No. 1704150EDU). Membranes were blocked with 5% non-fat dry milk (Bio-Rad, Cat. No. 1706404) in TBST (Tris-buffered saline with 0.1% Tween-20) for 1 h at RT.

Primary antibody incubation was performed overnight at 4 °C with Anti-DDDDK-Tag Rabbit mAb (1:5000 dilution, Abclonal, Cat. No. AE092) to detect RSPH4A-Flag fusion proteins. After washing with 1× TBST, membranes were incubated with HRP-conjugated Goat Anti-Rabbit IgG (H + L) secondary antibody (dilution 1:10,000, Abclonal, Cat. No. AS014) for 1 h at RT. Protein bands were visualized using the SuperSignal^TM^ West Pico PLUS Chemiluminescent Substrate (ThermoFisher Scientific, Cat. No. 34580) on a ChemiDoc^TM^ MP Imaging System (Bio-Rad, Cat. No. 12003154).

As loading controls, membranes were stripped using Restore^TM^ PLUS Western blot Stripping Buffer (ThermoFisher Scientific, Cat. No. 46430) and reprobed with Anti-GAPDH Mouse mAb (dilution 1:100,000, Abclonal, Cat. No. AC033). Band intensities were quantified using ImageJ v1.53t (National Institutes of Health) with background subtraction. Relative protein expression was normalized to GAPDH levels.

### 2.10 Statistical analysis

Data are presented as mean ± standard deviation (SD) from three independent experiments. Statistical significance was determined using unpaired two-tailed Student’s t-tests performed with GraphPad Prism v9.5.1 (GraphPad Software, Inc.). A *P* value < 0.05 was considered statistically significant. One-way ANOVA followed by Tukey’s *post hoc* test was used for multiple group comparisons where appropriate.

## 3 Results

### 3.1 Clinical diagnosis of the PCD patient

The proband was an 11-year-old female, the elder of twins (with a dizygotic twin sister), born preterm. The mother had an uneventful pregnancy, and the patient had no history of birth asphyxia or resuscitation at delivery. At approximately 2 months of age, she experienced cyanosis and tachypnea requiring hospitalization at a local hospital (specific treatment details unknown), with subsequent improvement and discharge. She had a history of dust mite allergy and allergic rhinitis. The proband presented to the Department of Pediatrics at Jiaxing Hospital of T.C.M. in May 2024 with complaints of recurrent cough and sputum production for 4 years, aggravated over the past week. Four years prior (at approximately 7 years of age), the patient developed recurrent cough with sputum, accompanied by nasal congestion and rhinorrhea, and was diagnosed with pneumonia on multiple occasions, requiring repeated hospitalizations at local hospitals (specific treatment details unknown). In August 2023, she was evaluated at a local hospital for cough and sputum, where chest CT revealed consolidation in the right middle lobe with mild focal bronchial dilation, and minimal inflammation in the left upper lobe. She was admitted for treatment and underwent bronchoscopy with bronchoalveolar lavage, which revealed abundant viscous secretions bilaterally and turbid lavage fluid; the diagnosis was bronchial mucosal inflammation. Treatment included nebulized medication and oral azithromycin dry suspension (5 mg/kg once daily). Following discharge, the patient continued to experience recurrent cough with yellow sputum and occasional rhinorrhea, without further follow-up or treatment. One week prior to admission, she developed paroxysmal cough with increased frequency and yellow sputum without apparent precipitant, accompanied by fever reaching 39.0 °C, which subsequently resolved spontaneously. She also experienced nasal congestion with purulent discharge, chest tightness without wheezing, and was admitted with a provisional diagnosis of pneumonia and possible asthma.

**FIGURE 1:**
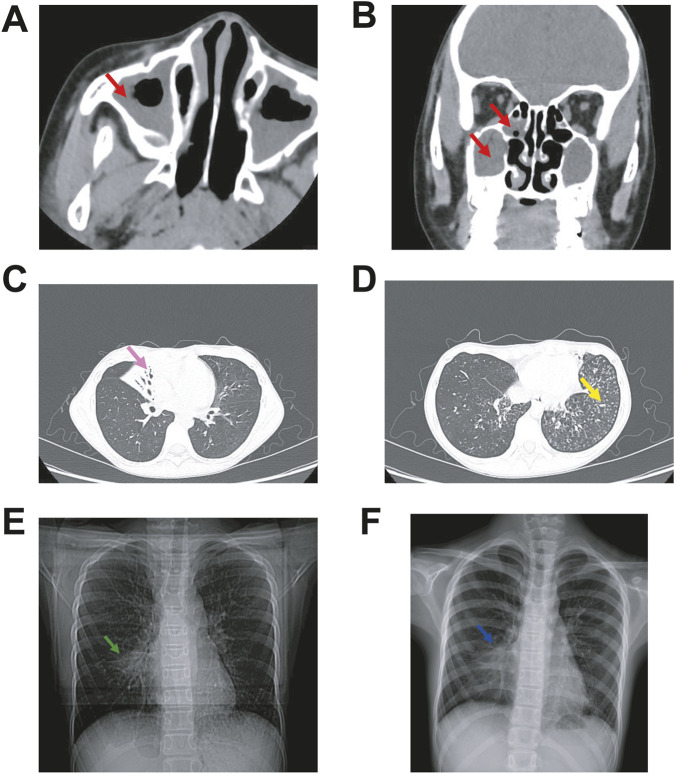
Paranasal sinus and chest computed tomography results. **(A)** and **(B)** Paranasal sinus CT results showed paranasal sinusitis, right inferior turbinate hypertrophy, and soft tissue thickening in the nasopharynx. Red arrow in **(A)** indicated mucosal thickening and increased density in the frontal sinus; Red arrow in **(B)** denoted mucosal thickening and increased density in the ethmoid and maxillary sinuses **(C,D)** Chest CT results demonstrated bilateral pulmonary infectious lesions with partial consolidation, focal bronchial dilation. Pink arrow in **(C)** presented atelectasis with consolidation in the right middle lobe, with visible bronchial dilation; Yellow arrow in **(D)** showed a “tree-in-bud” pattern in the left lung. **(E)** Coronal scout view of the chest CT showed patchy opacities with ill-defined margins in the right lower lung field adjacent to the mediastinum (indicated by the green arrow) **(F)** Chest radiography presented increased and blurred bronchovascular markings bilaterally in the proband. Multiple patchy, ill-defined opacities with diffuse distribution were observed in the left lower lung field. The blue arrow denoted the pathological features in the right lower lung field adjacent to the mediastinum were consistent with the findings from the coronal scout CT image.

**FIGURE 2:**
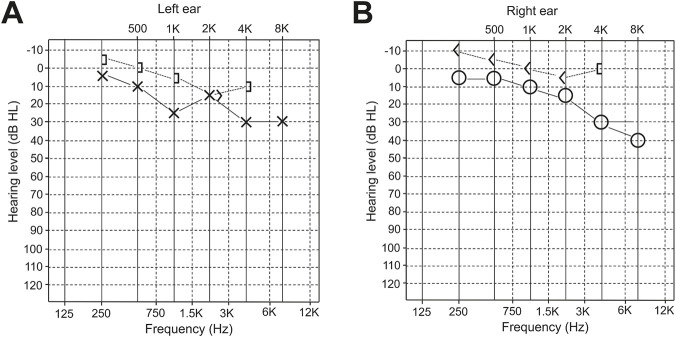
Bilateral audiometric evaluation results of the proband. **(A,B)** Audiometric evaluation revealed high-frequency hearing impairment in the left ear **(A)** and right ear **(B)** of the proband. Symbol definitions: ‘ × ’ indicates unmasked left ear during air conduction testing, ‘O’ denotes unmasked right ear during air conduction testing, ‘]’ presents masked left ear during bone conduction testing, ‘[’ presents masked right ear during bone conduction testing, ‘>’ indicates unmasked left ear during bone conduction testing, ‘<’ denotes unmasked right ear during bone conduction testing.

Comprehensive diagnostic workup of the proband was performed: (1) Physical examination revealed: height 149 cm, weight 32.5 kg, body temperature 36.3 °C, pulse 92 beats per minute, respiratory rate 25 breaths per minute, blood pressure 107/73 mmHg, oxygen saturation 96%, alert and oriented, mildly lethargic, skin without rash or petechiae, copious purulent nasal secretions, regular respiration without nasal flaring or retractions, erythematous pharynx, bilateral grade II tonsillar enlargement, coarse breath sounds bilaterally with moist rales and expiratory wheezing, regular heart rhythm without murmur, soft abdomen without tenderness, liver and spleen not palpable below costal margins, normal neurological examination, no digital clubbing; (2) Laboratory investigations showed complete blood count: white blood cells 9.08 × 10^9^/L, neutrophils percentage 59.9%, lymphocytes percentage 33.5%, eosinophils percentage 0.6%, hemoglobin 134.0 g/L, platelets 215 × 10^9^/L, high-sensitivity C-reactive protein (CRP) 4.66 mg/L; liver and renal function tests, cardiac enzymes, coagulation profile, procalcitonin, ferritin, and absolute lymphocyte subset counts were all unremarkable; Tuberculosis antibody, purified protein derivative (PPD) skin test, and *Mycoplasma* pneumoniae nucleic acid detection were negative, and blood culture was sterile; Immunoglobulin A was 2.32 g/L (reference range 0.53–2.04 g/L), immunoglobulin G 14.730 g/L (reference range 6.98–15.6 g/L), and immunoglobulin M 2.11 g/L (reference range 0.31–1.79 g/L); (3) Allergen testing revealed: house dust mite (Dermatophagoides pteronyssinus/farinae) 2.72 IU/mL (reference <0.35 IU/mL), with other allergens negative. Rhinovirus RNA qualitative detection: positive (+); (4) Paranasal sinus computed tomography (CT) demonstrated paranasal sinusitis, right inferior turbinate hypertrophy, and soft tissue thickening in the nasopharynx (Figures 1A,B); (5) Chest CT revealed bilateral pulmonary infectious lesions with partial consolidation, focal bronchial dilation, and minimal right-sided pleural effusion (Figures 1C,D). The coronal scout view of the chest CT revealed patchy opacities with ill-defined margins in the right lower lung field adjacent to the mediastinum (Figure 1E). Additionally, chest radiography showed increased and blurred bronchovascular markings bilaterally in the proband. Multiple patchy, ill-defined opacities with diffuse distribution were observed in the left lower lung field, and the pathological features in the right lower lung field adjacent to the mediastinum were consistent with the findings from the coronal scout CT image (Figure 1F); (6) Routine electrocardiography and echocardiography were unremarkable; (7) Pulmonary function testing indicated moderate mixed ventilatory dysfunction (FVC 51.3% predicted, FEV1 41.7% predicted); (8) Bronchodilator reversibility test was negative; (9) Fractional exhaled nitric oxide (FeNO) 11 ppb, nasal nitric oxide (FnNO) 19 ppb; (10) Audiometric evaluation revealed high-frequency hearing loss in the proband (Figures 2A,B). Although a sweat chloride test was not performed, comprehensive WES analysis excluded pathogenic or likely pathogenic variants in all known cystic fibrosis (CF, MIM #219700)-causing genes (such as *CFTR, TGFB1,* and *FCGR2A*). Despite significant phenotypic overlap between CF and PCD, the proband’s clinical presentation, notably the absence of pancreatic involvement and normal growth parameters, favored a diagnosis of PCD over CF.

**FIGURE 3:**
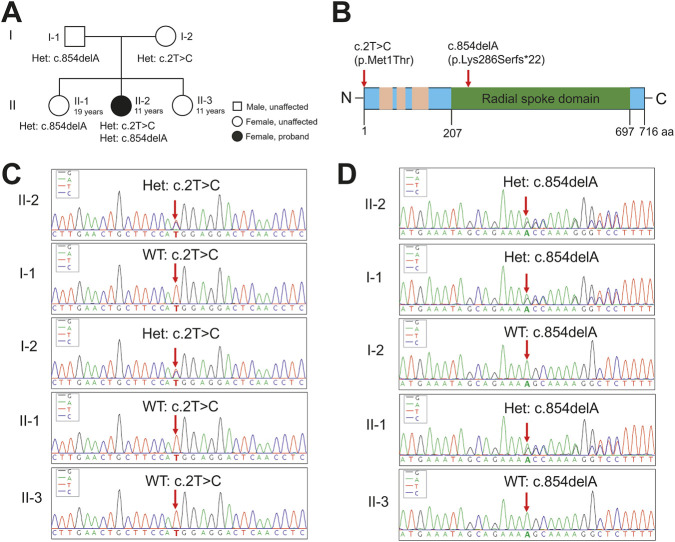
WES and Sanger sequencing identified biallelic RSPH4A variants strictly co-segregating with PCD in family. **(A)** Pedigree analysis of the PCD proband’s family **(B)** Schematic diagram of RSPH4A protein structure indicating the positions of c.2T>C and c.854delA variants. N indicates N-terminus, C indicates C-terminus, aa indicates amino acid, orange indicates low complexity sequence of RSPH4A protein, green indicates radial spoke domain sequence of RSPH4A protein **(C)** Sanger sequencing results showed that the proband (II-2) and her mother (I-2) carried the heterozygous c.2T>C variant, while the proband’s father (I-1), elder sister (II-1), and dizygotic twin sister (II-3) did not carry this missense variant **(D)** Sanger sequencing results showed that the proband (II-2), her father (I-1), and elder sister (II-1) carried the heterozygous c.854delA variant, while the proband’s mother (I-2) and dizygotic twin sister (II-3) did not carry this frameshift deletion.

The family pedigree consisted of five members: the affected proband (II-2), her 19-year-old healthy sister (II-1), her dizygotic twin sister (II-3), the unaffected father (I-1), and the unaffected mother (I-2) (Figure 3A). No other family members reported symptoms suggestive of PCD. The parents denied consanguinity, and the family originated from Zhejiang Province, China.

### 3.2 Identification of biallelic *RSPH4A* variants by whole exome sequencing

To elucidate the genetic etiology of this PCD case, we performed WES on the proband’s genomic DNA. WES generated 15.31 Gb raw data, and achieved a mean coverage depth of 167.06× across the target regions, with 99.57% of bases covered at ≥ 20× depth. Bioinformatics analysis identified approximately 73,201 single nucleotide variants and indels after quality filtering.

Following our variant filtering strategy focusing on PCD-associated genes, we identified compound heterozygous variants in the *RSPH4A* gene (NM_001010892.3): a start codon variant c.2T>C (p.Met1Thr) and a frameshift deletion c.854delA (p.Lys286Serfs*22) in the radial spoke domain (Figure 3B). The c.854delA variant was absent from population databases including gnomAD, ExAC, and the 1000 Genomes Project, whereas the c.2T>C variant was absent from ExAC and the 1000 Genomes Project, but present in gnomAD at an allele frequency of 3.2 × 10^−5^ (6.0 × 10^−4^ in the gnomAD East Asian population). According to the American College of Medical Genetics and Genomics (ACMG) guidelines, both *RSPH4A* (NM_001010892.3): c.2T>C (p.Met1Thr) and *RSPH4A* (NM_001010892.3): c.854delA (p.Lys286Serfs*22) were classified as pathogenic (evidence: PM2_Supporting, PVS1, PM3).

Sanger sequencing was performed to validate these variants and determine their segregation within the family. The results confirmed that the proband carried both variants in trans configuration: the c.2T>C variant was inherited from the mother, while the c.854delA variant was inherited from the father (Figures 3C,D). The elder sister (II-1) was heterozygous for the c.854delA variant inherited from the father, while both c.2T>C and c.854delA variant was absent from the twin sister (II-3) sample (Figures 3C,D). Neither parent carried both variants, consistent with autosomal recessive inheritance. The strict co-segregation of the *RSPH4A* biallelic variants with the disease phenotype in this family strongly supports the pathogenicity of these variants as the genetic cause of PCD in the proband. The absence of these variants in population databases and their predicted damaging effects on protein function further substantiate their clinical significance.

### 3.3 Functional and structural analysis of the biallelic *RSPH4A* variants

**FIGURE 4:**
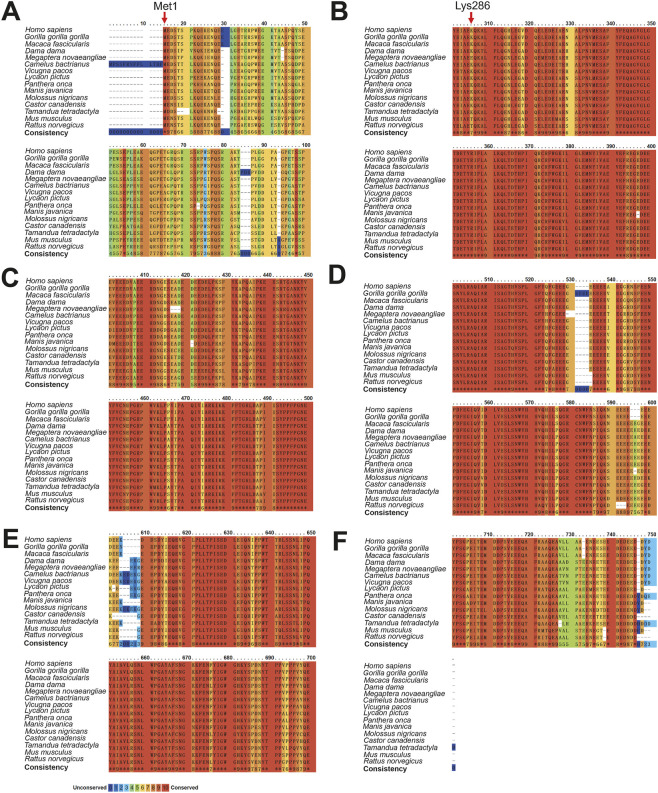
Multiple sequence alignment of RSPH4A protein across 15 species and conservation analysis of Lys286 and neighboring residues. **(A)** Alignment of N-terminal sequences (1-82 aa) of RSPH4A protein from 15 species (including *Homo sapiens*, *Gorilla gorilla gorilla*, *Macaca fascicularis*, *Mus musculus*, *Rattus norvegicus*, *Dama*, *Megaptera novaeangliae*, *Lycaon pictus*, *Molossus nigricans*, *Panthera onca*, *Tamandua tetradactyla*, *Camelus bactrianus*, *Castor canadensis*, *Manis javanica*, and *Vicugna pacos*), with red arrow indicating the methionine residue (Met1) encoded by the initiation codon **(B–F)** Alignment of radial spoke domain sequences (281-716 aa) of RSPH4A protein from 15 species, with red arrow indicating the Lys286 residue position. The online tool PRALINE was used to perform the analysis. Values ranging from 0 to 10 represent the conservation degree of each amino acid residue.

The c.2T>C variant located at the initiation codon (ATG→ACG) disrupts the canonical start codon for translation initiation (Figure 4A). This variant is predicted to abolish normal translation initiation from the first methionine. In silico analysis using SIFT (damaging), PolyPhen-2 (probably damaging), and MutationTaster (disease-causing) consistently predicted this variant to be deleterious. Potential consequences include NMD if translation fails to initiate, or translation initiation from downstream alternative start codons producing N-terminally truncated proteins lacking critical functional domains.

The c.854delA single nucleotide deletion in exon 2 causes a frameshift starting at lysine 286, replacing the native amino acid sequence with 21 aberrant residues (Ser-Lys-Arg-Leu-Phe-Phe-Ser-Arg-Asp-Ile-Trp-Lys-Glu-Leu-Thr-Lys-Asn-Trp-Lys-Met-Lys) before encountering a premature termination codon at position 307. This results in a truncated protein of 306 amino acids compared to the wild-type 716 amino acids, eliminating approximately 57% of the C-terminal protein sequence including the majority of the radial spoke head domain.

Cross-species protein sequence alignment using PRALINE revealed that the radial spoke head domain (i.e. 207-697 aa), particularly the region surrounding Lys286, is highly conserved across 15 vertebrate species spanning mammals from primates (*H. sapiens*, *Gorilla gorilla gorilla*, *M. fascicularis*) to rodents (*M. musculus*, *R. norvegicus*), carnivores (*L. pictus*, *P. onca*), cetaceans (*M. novaeangliae*), bats (*M. nigricans*), and other diverse mammals (*Dama*, *T. tetradactyla*, *C. bactrianus*, *C. canadensis*, *M. javanica*, *V. pacos*) (Figures 4B–F). The Lys286 residue and surrounding sequence (aa 281–380) showed >94.8% identity across all analyzed species (Figure 4B), underscoring the critical functional importance of this region.

**FIGURE 5:**
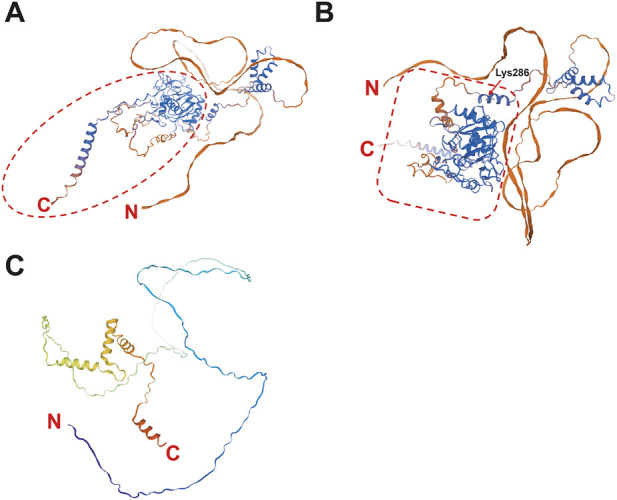
Structural modeling of wild-type and mutant RSPH4A proteins. **(A)** and **(B)** Structural modeling results of wild-type RSPH4A protein shown from different perspectives. The human RSPH4A protein sequence (NP_001010892.1, 716 aa) was used for structural modeling analysis, with red arrow indicating the Lys286 amino acid residue position **(C)** Structural modeling result of mutant RSPH4A protein (p.Lys286Serfs*22). The truncated human RSPH4A protein sequence (306 aa) was used for structural modeling analysis, and the results showed loss of almost the entire radial spoke domain sequence (307-697 aa) and C-terminal sequence, with disruption of secondary and tertiary structures. N indicates N-terminus, C indicates C-terminus. The online tool SWISS-MODEL was used for structural modeling.

Protein structure modeling using SWISS-MODEL demonstrated that wild-type RSPH4A forms an extended structure comprising an N-terminal domain (aa 1–206), a radial spoke head domain (aa 207–697), and a C-terminal tail (aa 698–716) (Figures 5A,B). The c.854delA truncation eliminates almost the entire C-terminal portion of the radial spoke head domain (aa 286–697) and the complete C-terminal tail (Figure 5C). Structural analysis revealed that the truncated protein lacks critical α-helical bundles and β-sheet structures required for proper radial spoke assembly and interaction with the central pair apparatus. The frameshift-derived C-terminal peptide (21 aa) is predicted to be unstructured and unlikely to compensate for the lost functional domains.

### 3.4 Experimental validation of *RSPH4A*: c.2T>C and *RSPH4A*: c.854delA function

To experimentally validate the functional consequences of the identified variants, we performed *in vitro* expression studies using HEK293T cells. Wild-type (WT) *RSPH4A* cDNA and mutant constructs harboring c.2T>C or c.854delA were cloned into the pIRES2-EGFP expression vector, enabling simultaneous expression of the target protein and EGFP reporter.

**FIGURE 6:**
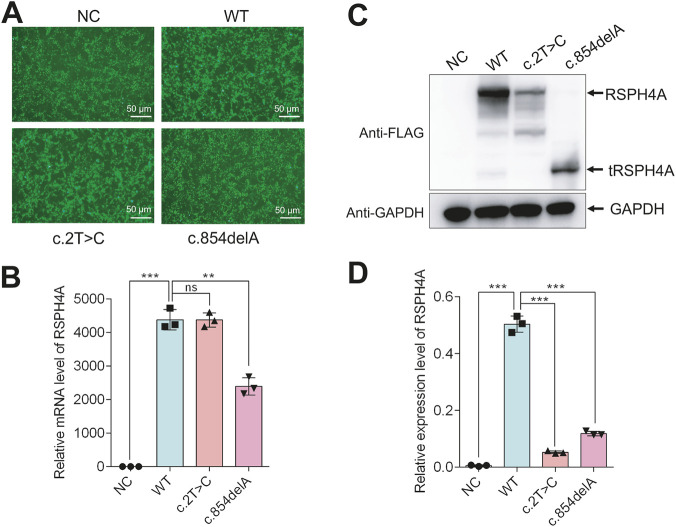
The c.2T>C and c.854delA variants significantly reduce RSPH4A protein expression levels. **(A)** Fluorescence microscopy observation of EGFP expression showed comparable transfection efficiencies of empty vector (negative control, NC), wild-type RSPH4A (WT), and two mutant RSPH4A constructs (c.2T>C and c.854delA) in HEK-293T cells. Scale bar: 50 μm **(B)** qRT-PCR results showing RSPH4A mRNA expression levels in four groups of HEK-293T cells: NC, wild-type RSPH4A (WT), and two mutant RSPH4A constructs (c.2T>C and c.854delA), with GAPDH as the internal reference gene **(C)** Western blotting results showing RSPH4A protein (M.W. 80.73 kDa) expression levels in four groups of HEK-293T cells: NC, wild-type RSPH4A (WT), and two mutant RSPH4A constructs (c.2T>C and c.854delA), tRSPH4A: truncated RSPH4A (M.W. 34.23 kDa), with GAPDH as the internal reference protein **(D)** Bar graph showing RSPH4A protein expression levels in four groups of HEK-293T cells: NC, WT, c.2T>C, and c.854delA. **(A–D)** Three biological replicates were performed for each group, and all samples were analyzed using one-way ANOVA with Tukey’s *post hoc* test. ns: not significant. **: *P* < 0.01, ***: *P* < 0.001.

Fluorescence microscopy examination 48 h post-transfection confirmed robust EGFP expression in all four experimental groups (i.e., negative control, WT, c.2T>C, and c.854delA) (Figure 6A), indicating comparable transfection efficiencies across groups (approximately 75%–80% transfection rate). This ensured that any observed differences in target gene expression were attributable to variant effects rather than differential transfection efficiency.

Quantitative RT-PCR (qRT-PCR) analysis revealed that compared to the negative control (NC) group, all three constructs (WT, c.2T>C, and c.854delA) showed significant overexpression of *RSPH4A* mRNA (*P* < 0.001 for all), confirming successful plasmid delivery and transcription. Notably, comparison with the WT group showed that the c.2T>C mutant exhibited comparable mRNA levels (*P* > 0.05) (Figure 6B), suggesting that the start codon mutation does not trigger NMD, likely because the premature termination codon is located at the extreme 5′ end of the transcript. In contrast, the c.854delA mutant showed significantly reduced mRNA expression (approximately 55% of WT levels, *P* < 0.01) (Figure 6B), consistent with NMD triggered by the premature termination codon at position 307.

Western blotting using anti-FLAG antibody detected the expected full-length RSPH4A protein (M.W. approximately 80.73 kDa) in the WT group, which was absent in the NC group. The c.2T>C mutant showed dramatically reduced protein expression (approximately 10.4% of WT levels, *P* < 0.001) despite normal mRNA levels, demonstrating that disruption of the start codon severely impairs translation initiation. This confirms that c.2T>C is a loss-of-function variant at the translational level.

For the c.854delA mutant, Western blotting detected a truncated protein (tRSPH4A, M.W. approximately 34.23 kDa) corresponding to the predicted 306-amino acid product. However, the expression level of this truncated protein was significantly reduced compared to WT (approximately 23.5% of WT, *P* < 0.001), indicating decreased protein stability and likely increased degradation of the misfolded truncated protein. The truncated protein lacks the C-terminal radial spoke head domain and is therefore expected to be non-functional.

Collectively, these results demonstrate that both *RSPH4A* variants are loss-of-function mutations through distinct molecular mechanisms: c.2T>C disrupts translation initiation causing severe protein depletion despite intact mRNA, while c.854delA triggers NMD and produces an unstable truncated protein lacking essential functional domains. The compound heterozygous state results in complete absence of functional RSPH4A protein, explaining the severe PCD phenotype observed in the proband. These experimental findings confirm the pathogenicity of the identified variants and establish the molecular basis for disease development in this patient.

## 4 Discussion

PCD represents a significant diagnostic challenge in clinical genetics due to its extensive genetic heterogeneity, with pathogenic variants identified in over 50 genes encoding various ciliary components (Keicho et al., 2023; Horani and Ferkol, 2018; Black et al., 2024). The diagnostic complexity is further compounded by the lack of a single gold-standard test, requiring integration of clinical phenotyping, nNO measurement, HSVMA, TEM, and genetic testing (Mirra et al., 2017; Shapiro et al., 2016; Keicho et al., 2023; Snijders et al., 2014). Despite advances in next-generation sequencing, a substantial proportion of clinically diagnosed PCD patients remain genetically undiagnosed, with reported diagnostic yields ranging from 30% to 76% depending on the population and testing methodology (Keicho et al., 2023; Fleming et al., 2024; Wheway et al., 2021). A particularly vexing problem in PCD genetics is the interpretation of novel variants, especially those with atypical molecular consequences such as start codon mutations and frameshift variants in genes like *RSPH4A* (Wang et al., 2022). The ACMG guidelines for variant classification provide a framework, but functional validation remains essential for establishing pathogenicity, particularly for variants of uncertain significance (Richards et al., 2015). The increasing identification of novel variants through WES has outpaced our ability to functionally characterize them, creating a bottleneck that limits accurate genetic counseling and prenatal diagnostic capabilities for affected families.

This study reports the identification and functional characterization of two novel *RSPH4A* variants in a Chinese PCD patient: c.2T>C (p.Met1Thr) and c.854delA (p.Lys286Serfs*22). Our findings expand the mutational spectrum of *RSPH4A*-related PCD and provide important insights into the molecular mechanisms underlying radial spoke head protein dysfunction. The c.2T>C variant represents a rare class of start codon mutation that disrupts canonical translation initiation, while c.854delA introduces a premature termination codon within the critical radial spoke head domain. Previous studies revealed that *RSPH4A* mutations cause PCD with distinctive central-microtubular-pair abnormalities (Castleman et al., 2009; De Jesus-Rojas et al., 2023), and subsequent work demonstrated that RSPH4A is essential for assembly of all three radial spoke heads (RS1, RS2, and RS3) in mouse motile cilia (Yoke et al., 2020). Our functional studies align with these observations and provide experimental evidence that both variants result in substantial decrease in functional RSPH4A protein, consistent with the severe clinical phenotype observed in the proband. Notably, the strict co-segregation of these variants with the disease phenotype in this family, combined with their absence from population databases and predicted damaging effects, satisfies criteria for definitive pathogenicity according to ACMG guidelines (Richards et al., 2015). This study contributes to the growing body of evidence supporting the critical role of radial spoke head proteins in ciliary motility and establishes a framework for functional validation of *RSPH4A* variants that can be applied to future cases.

The functional characterization of these *RSPH4A* variants reveals distinct molecular mechanisms of protein dysfunction with important implications for disease pathogenesis and clinical management. The c.2T>C (p.Met1Thr) variant disrupts the canonical initiation codon, preventing normal translation initiation. Our qRT-PCR data demonstrated comparable mRNA levels between wild-type and c.2T>C constructs, indicating that this variant does not trigger NMD, likely because the premature termination codon is located at the extreme 5′ end of the transcript (Karousis et al., 2021; Sun and Chen, 2023). However, Western blotting revealed dramatically reduced protein expression, demonstrating that the primary consequence is translational failure. This finding is consistent with the observation that start codon mutations can lead to either complete absence of protein production or translation initiation from downstream alternative start codons, potentially producing N-terminally truncated proteins (Zhang and Xie, 2023; Kochetov et al., 2005). For the c.2T>C variant, the near-complete absence of detectable protein indicates that translation initiation is severely impaired. It is possible that alternative downstream initiation sites may be utilized; however, any protein products generated from such sites would likely be N-terminally truncated and potentially unstable or subject to rapid degradation, though this remains to be experimentally validated. Future studies should use ribosome profiling and pulse-chase labeling experiments to directly assess whether alternative translation initiation occurs in the c.2T>C mutant and to characterize the stability of any truncated protein products that may be generated.

The c.854delA (p.Lys286Serfs*22) variant introduces a frameshift at codon 286, resulting in a truncated protein of 306 amino acids with a novel C-terminal peptide of 21 residues before premature termination. This truncation eliminates approximately 57% of the C-terminal protein sequence, including the majority of the radial spoke head domain that is essential for proper assembly and function (Zhao et al., 2021). Our protein sequence alignment across 15 vertebrate species demonstrated that the affected region is highly conserved, with >94.8% identity in the radial spoke head domain, underscoring its functional importance (Grossman-Haham et al., 2021; Gui et al., 2021). Structural modeling using SWISS-MODEL (Waterhouse et al., 2018) predicted that the truncated protein lacks critical α-helical bundles and β-sheet structures required for proper radial spoke assembly and interaction with the central pair apparatus. Furthermore, our experimental data showed that c.854delA triggers NMD, reducing mRNA levels to approximately 55% of wild-type, and produces an unstable truncated protein at significantly reduced levels. These findings are consistent with recent cryo-electron tomography studies demonstrating that RSPH4A is a generic component essential for assembly of all three radial spoke heads (Zhao et al., 2021; Grossman-Haham et al., 2021; Gui et al., 2021; Zheng et al., 2021).

The proband’s clinical phenotype aligns with the expected consequences of complete RSPH4A deficiency. Early manifestations included cyanosis and tachypnea at approximately 2 months of age, consistent with neonatal respiratory distress. During childhood (from approximately 7 years of age onward), the patient developed recurrent respiratory tract infections (manifesting as recurrent cough, sputum production, and fever), rhinosinusitis, focal bronchial dilation, pneumonia, and hearing impairment secondary to chronic otitis media. This chronological progression—from early infantile respiratory distress to progressive chronic suppurative lung disease and associated complications during childhood—is characteristic of the natural history of untreated PCD. Notably, the absence of laterality defects in *RSPH4A*-related PCD, as observed in our patient, is explained by the 9 + 0 configuration of nodal cilia which lack the central pair apparatus affected by radial spoke dysfunction (Castleman et al., 2009). The identification of these biallelic loss-of-function variants has immediate clinical utility for genetic counseling of the affected family. Preimplantation genetic diagnosis (PGD) and prenatal testing can now be offered for future pregnancies, enabling prevention of PCD recurrence in this family. Furthermore, the functional validation strategy employed in this study establishes a template for interpreting novel *RSPH4A* variants, facilitating accurate molecular diagnosis and appropriate clinical management for other PCD patients.

Several limitations of this study should be acknowledged. First, our functional studies were performed in HEK293T cells, which do not possess motile cilia, rather than in airway epithelial cells or patient-derived ciliated cells. While this heterologous expression system allowed quantitative assessment of mRNA stability and protein expression, it cannot fully recapitulate the complex cellular environment of respiratory cilia or assess the impact of these variants on ciliary beat pattern and waveform. Future studies should utilize air-liquid interface (ALI) cultures of patient-derived nasal epithelial cells to directly examine ciliary ultrastructure and motility. Second, we did not perform rescue experiments in animal models such as zebrafish or mouse *Rsph4a* knockout models in the current study. Next, we plan to utilize zebrafish and mouse *Rsph4a* knockout models to perform rescue experiments, which would provide definitive *in vivo* evidence that the identified variants cause ciliary dysfunction. Third, we did not perform HSVMA on patient-derived nasal epithelial cells to directly assess ciliary beat pattern and waveform. HSVMA is considered a key diagnostic modality for PCD, and its absence represents a limitation of our current study. We plan to perform HSVMA analysis in follow-up studies. Fourth, our study focused on two specific variants and did not comprehensively assess the full spectrum of *RSPH4A* mutations; a larger cohort study would be valuable for establishing genotype-phenotype correlations and identifying potential modifier genes. Finally, we did not evaluate potential therapeutic interventions, such as read-through agents for nonsense mutations or gene therapy approaches, which represent promising future directions for PCD treatment. Ongoing work in our laboratory aims to address these limitations by establishing patient-specific induced pluripotent stem cell (iPSC) lines for disease modeling and exploring CRISPR-based gene correction strategies. Despite these limitations, our study provides strong evidence for the pathogenicity of these novel *RSPH4A* variants and provides a basis for informed genetic counseling and potential future therapeutic interventions in PCD.

## 5 Conclusion

We identified two novel biallelic *RSPH4A* loss-of-function variants—c.2T>C (p.Met1Thr) and c.854delA (p.Lys286Serfs*22)—causing PCD in a Chinese patient. Functional studies revealed distinct molecular mechanisms: the start codon mutation abolishes translation initiation, while the frameshift deletion triggers nonsense-mediated mRNA decay and produces unstable truncated protein. This study expands the *RSPH4A* mutational spectrum, establishes a robust framework for variant interpretation, and provides a basis for informed genetic counseling for the PCD-affected family. Our findings demonstrate how comprehensive functional validation bridges genetic discovery and clinical application, ultimately improving outcomes for PCD patients.

## Data Availability

The original contributions presented in the study are included in the article/supplementary material, further inquiries can be directed to the corresponding author.
